# Supplementary material for: From Serendipity to Rational Identification of the 5,6,7,8-Tetrahydrobenzo[4,5]thieno[2,3-d]pyrimidin-4(3H)-one Core as a New Chemotype of AKT1 Inhibitors for Acute Myeloid Leukemia
Source: Pharmaceutics. 2022 Oct 26;14(11):2295. doi: 10.3390/pharmaceutics14112295 (PMC9698716; doi:10.3390/pharmaceutics14112295)
Supplement: Supplementary file 1 [file pharmaceutics-14-02295-s001.zip › pharmaceutics-1917099-supplementary.pdf]

Supplementary Materials

From Serendipity to Rational Identification of the 5,6,7,8-tetrahydrobenzo[4,5]thieno[2,3-*d*]pyrimidin-4(3*H*)-one Core as a New Chemotype of AKT1 Inhibitors for Acute Myeloid Leukemia

Andrea Astolfi,[1†] Francesca Milano,[2†] Deborah Palazzotti,[1†] Jose Brea,[3] Maria Chiara Pismataro,[1] Mariangela Morlando,[1] Oriana Tabarrini,[1] Maria Isabel Loza,[3] Serena Massari,[1] Maria Paola Martelli,[2] and Maria Letizia Barreca[1,*]

[1] Department of Pharmaceutical Sciences, "Department of Excellence 2018-2022", University of Perugia, 06123, Perugia, Italy

[2] Department of Medicine and Surgery, Hematology, Center for Research in Hemato-Oncology (CREO), University of Perugia, Perugia, Italy

[3] CIMUS Research Center, University of Santiago de Compostela, 15782, Santiago de Compostela, Spain

To whom corresponding should be addressed: maria.barreca@unipg.it

Table of Contents

1. Supporting Figures and Tables
   Figure S1
   Figure S2
   Figure S3
   Figure S4
   Figure S5
   Figure S6
   Figure S7
   Figure S8
   Table S1
   Table S2
   Table S3
   Table S4
2. References

1. Supporting Figures and Tables

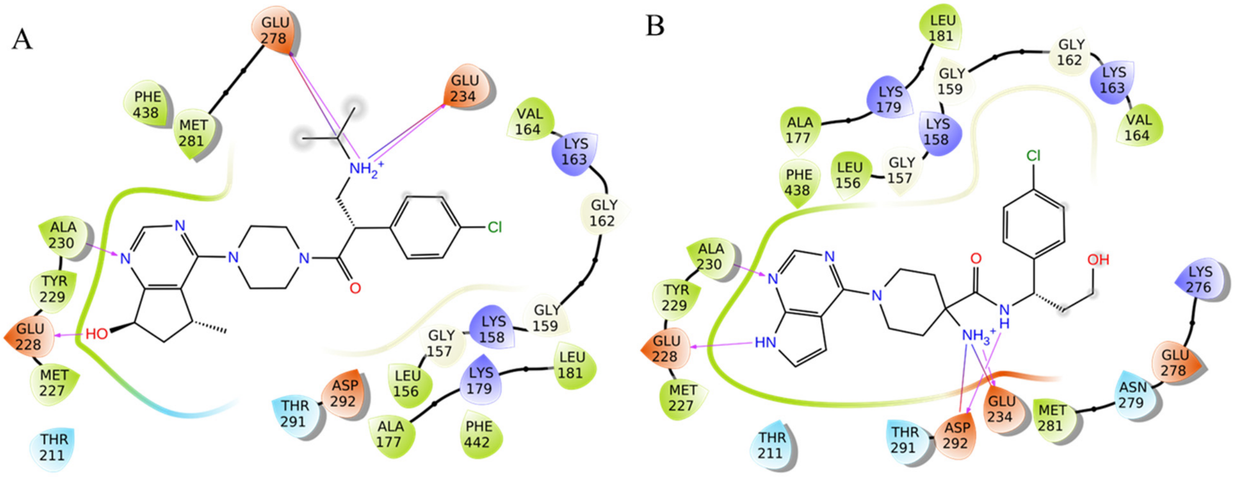

Capivasertib
IC_{50}: 3 nM
LE: 0.36

Ipatasertib
IC_{50}: 5 nM
LE: 0.36

Figure S1. Schematic 2D representation of the protein-ligand interactions established by the advanced co-crystallized inhibitors **capivasertib** (A) and **ipatasertib** (B) within the AKT1 ATP-binding pocket. The related IC_{50} (compound concentration necessary to reduce by 50% the enzymatic activity of AKT1) and ligand efficiency (LE= 1.4pIC_{50}/N, where N is the number of heavy atoms) values for the two AKT1 small molecule inhibitors are reported as well. Protein residues are color-coded as follow: red, charged residue (negative); blue, charged residue (positive); green, hydrophobic residue; cyan, polar residue.

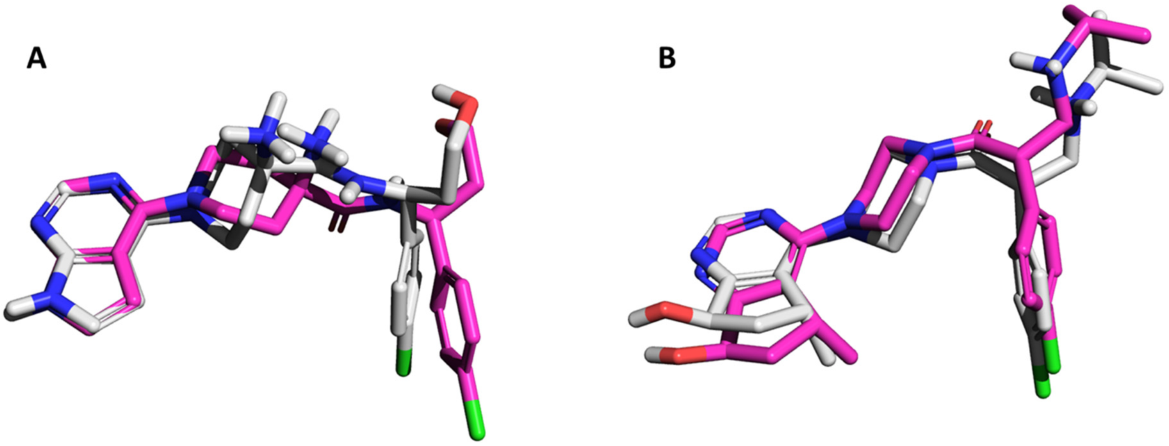

Figure S2. Superimposition of the experimental (white) and docking predicted (magenta) poses for **capivasertib** (A; RMSD = 2.46 Å) and **ipatasertib** (B, RMSD = 1.07 Å). The RMSD values were calculated on the ligand heavy atoms.

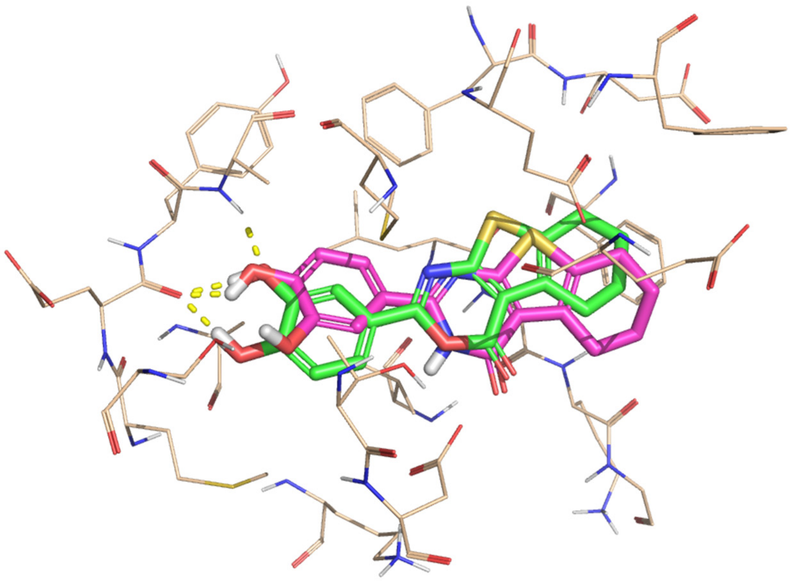

Figure S3. Superimposition of the pyrimidin-4(3*H*)-one derivative **T126** and the oxazin-4-one derivative **T125** binding modes.

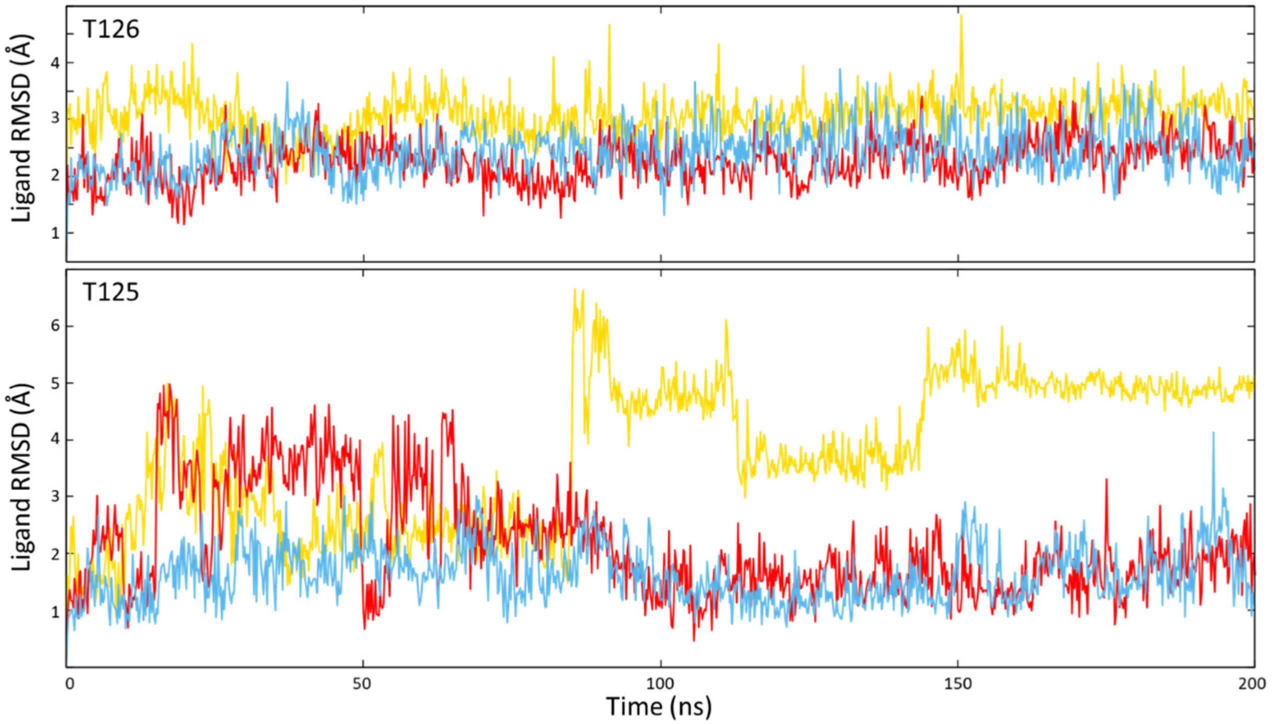

Figure S4. Ligand root mean square deviation (RMSD) computed for the three MD replicas on the **T126**-AKT1 and **T125**-AKT1 complexes. The ligand RMSD values were calculated as distance between ligand heavy atoms of each frame with respect to the ligand position on frame 0. Yellow line, replica 1; red line, replica 2; cyan line, replica 3.

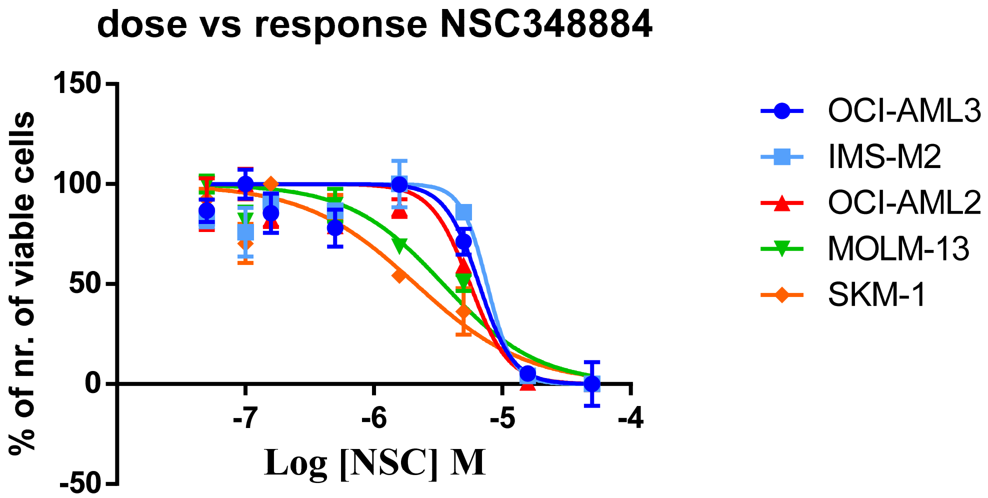

Figure S5. Dose-response curves for the reference compound NSC348884 (positive control) as generated by CellTiter Blue assay for the human AML cell lines OCI-AML3, IMS-M2, OCI-AML2, MOLM-13 and SKM-1. Calculated IC_{50} correspond to 6.6, 7.6, 5.6, 3.4 and 2.1 μM, respectively.

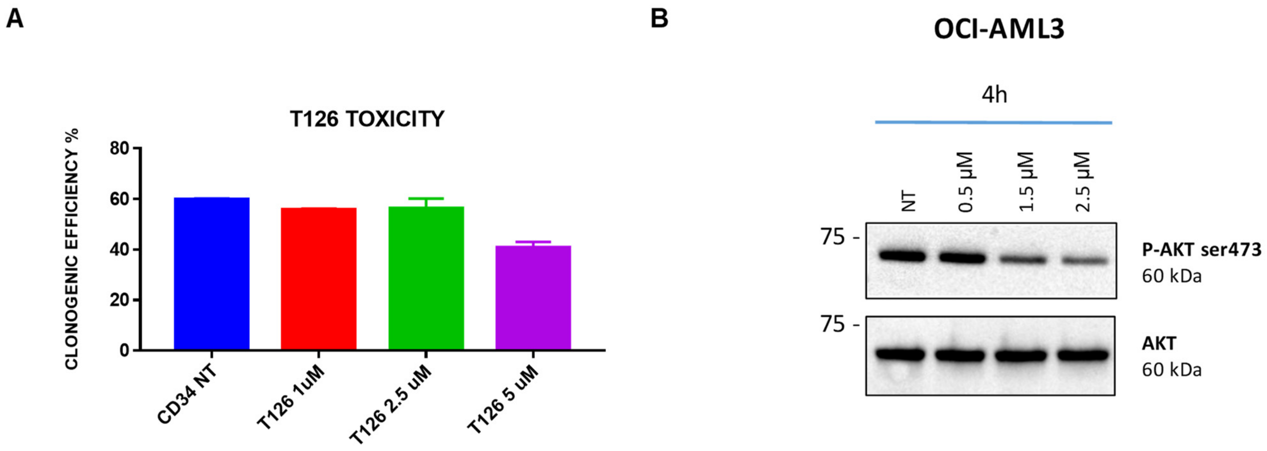

Figure S6. A) CFU assay on normal donor CD34+ hematopoietic stem/progenitor cells (HSPC). Represented are the number of cells that gave rise to colonies after 2 weeks of culture out of the total number of cells per well (Clonogenic Efficiency %).
B) Effects of **T126** at 0.5, 1.5 and 2.5 μM dose on phosphorylation of AKT at 4 hours (4h) in OCI-AML3. Total AKT expression levels were used as control for protein loading, as indicated. Representative results of at least three independent experiments are shown.

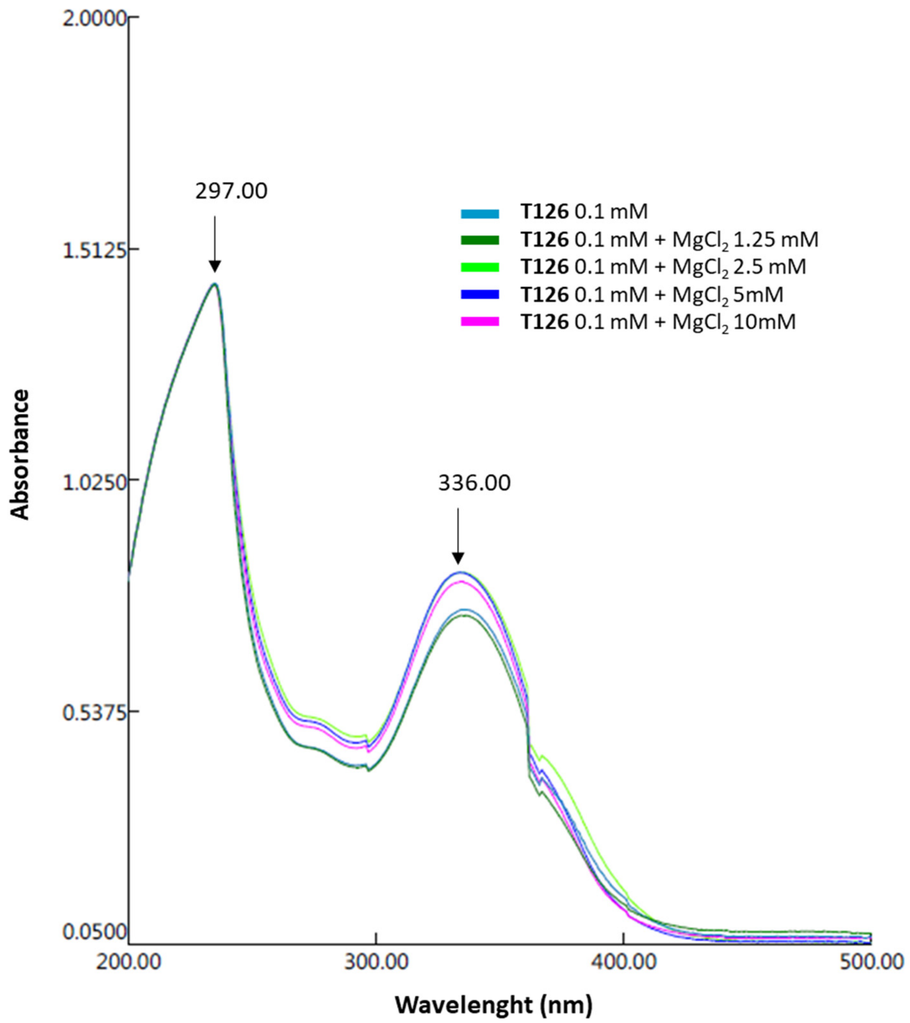

Figure S7: UV-vis spectra of compound **T126** measured alone and in presence of increasing concentrations of $MgCl_2$.

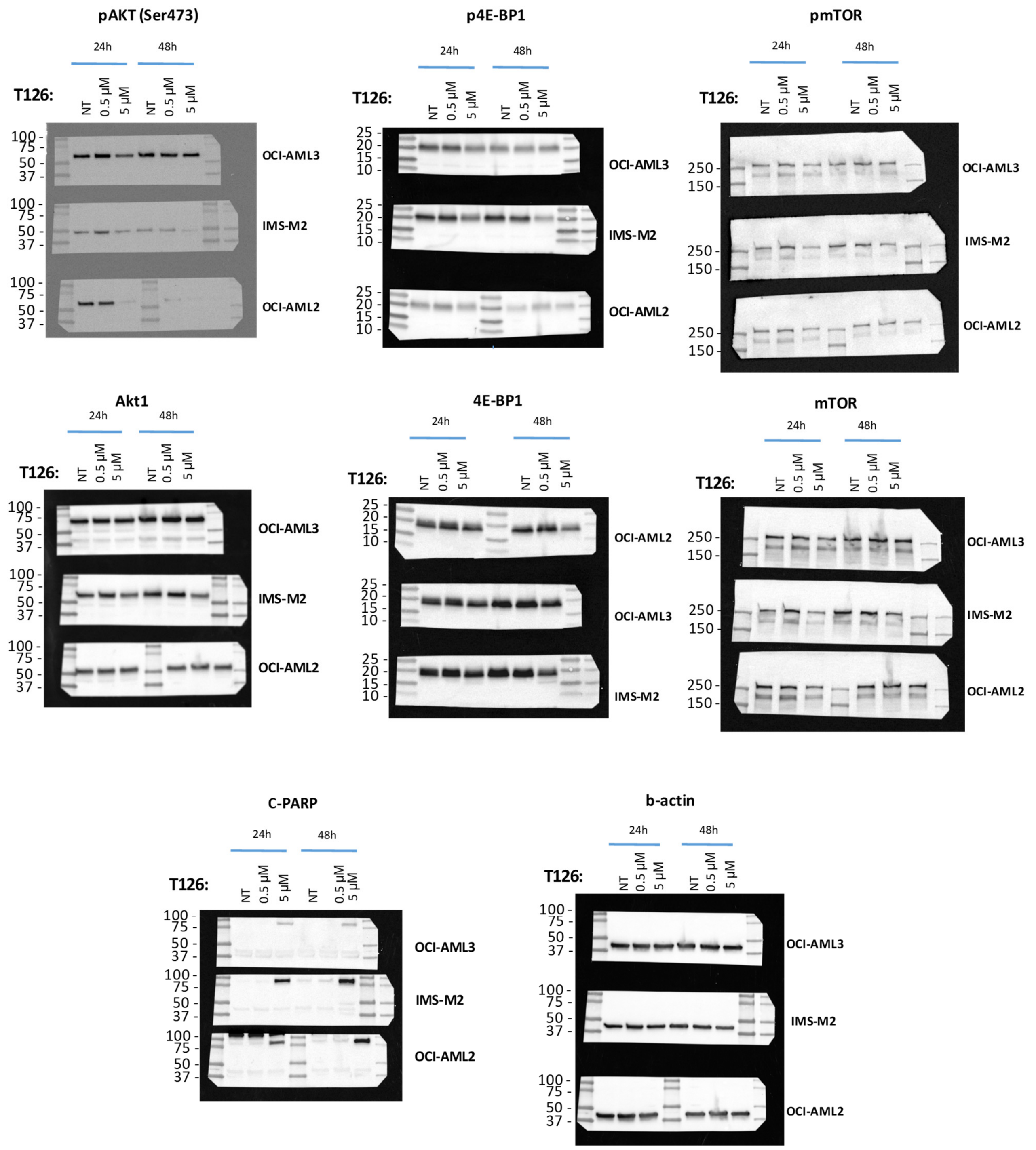

Figure S8. Uncropped full-length pictures of western blotting membranes related to Figure 4C, as directly acquired at Biorad Chemidoc (Biorad). Molecular weights (MW) corresponding to the marker used are indicated (in kDalton) on the left of each image. The name of the cell line to which experiment refers is indicated on the right of each image. The protein for which the blotting corresponds and the experimental conditions are indicated on the top of each image.

Table S1: Summary of the docking results. For each compound, we have reported the relative computed binding-free energy (kcal/mol) obtained by Prime MM–GBSA and the presence (highlighted by the "X" symbol) of the predicted interaction between the compound and the key ATP binding site residues Ala230 and/or Glu228.

Compound	*MMGBSA dG Bind*	*Ala230*	*Glu228*
**T101**	-43.341	X	X
**T116**	-49.066	X	X
**T117**	-47.474	X	X
**T124**	-49.455		X
**T125**	-58.274		X
**T126**	-48.516	X	X
**T127**	-55.439		X
**T128**	-54.023		X
**T129**	-47.709		X
**T133**	-42.455		X
**T134**	-44.709		X
**T159**	-42.439	X	X

Table S2: Chemical structure and biological activity for the selected in-house virtual hits against AKT1. The initial compound **T187** is reported as well. The compounds are listed according to the FTrees global similarity score.

Compound	%inh[a]	IC50 (μM)[c]	Ki (μM)[d]	Ftrees similarity[f]
**T187**	56[b]	11.4	4.19	**Query structure**
**T159 (ref.[32])**	94	2.12	1.91	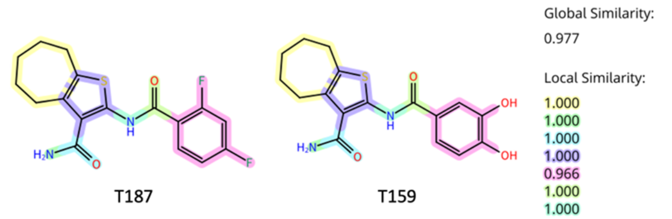
**T134 (ref. [32])**	31	ND[e]	ND[e]	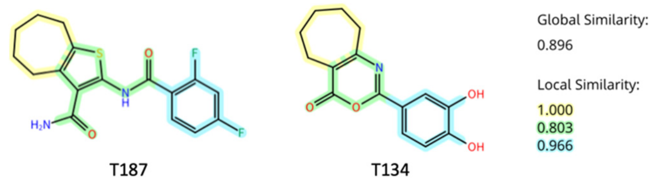
**T124 (ref. [34])**	>60	30.9	ND[e]	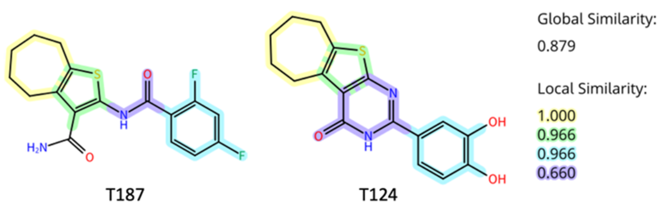
**T126 (ref. [34])**	87	1.99	0.41	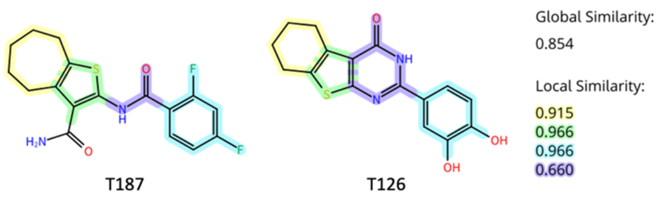
**T116 (ref. [32])**	2	ND[e]	ND[e]	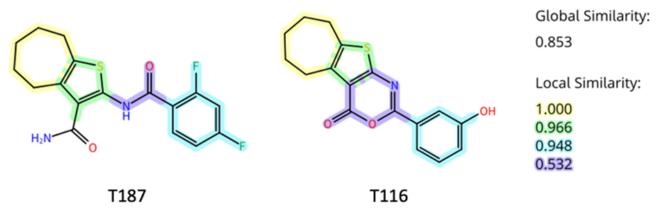
**T117 (ref. [32])**	1	ND[e]	ND[e]	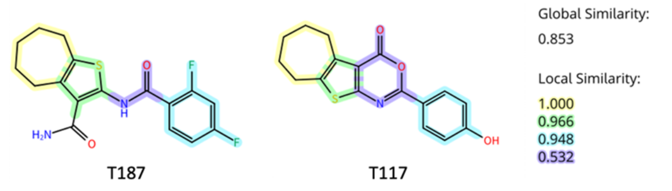

Table S2: Continued.

Compound	%inh[a]	IC50 (μM) [c]	Ki (μM) [d]	Ftrees similarity[f]
**T125 (ref. [32])**	9	ND[e]	ND[e]	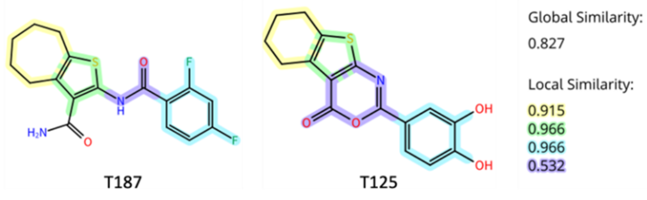
**T128 (ref. [34])**	78	26.6	ND[e]	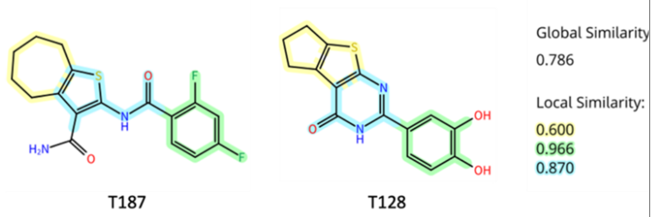
**T127 (ref. [32])**	43	ND[e]	ND[e]	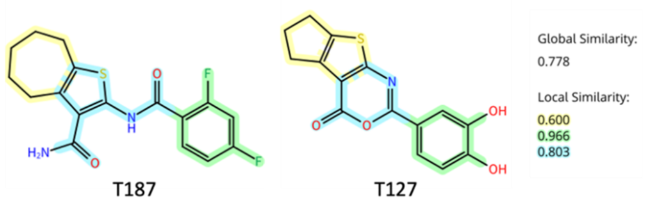
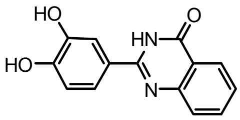 **T133 (ref [32])**	36	ND[e]	ND[e]	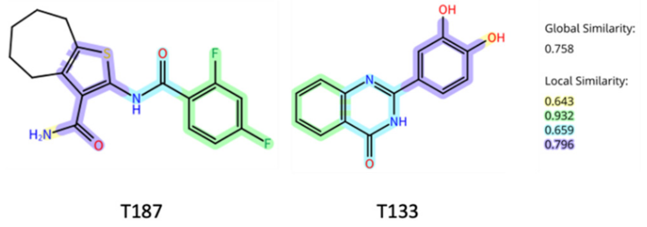
**T101 (ref. [35])**	95	1.62	0.24	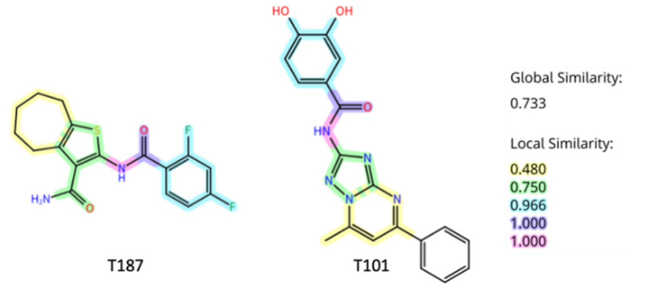
**T129 (ref. [32])**	8	ND[e]	ND[e]	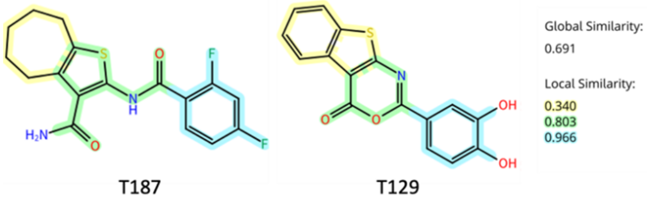

[a] The percentage of inhibition of AKT1 activity employing a 10 μM compound solution. Only compounds that were able to inhibit the kinase activity by a percentage ≥60% were further analysed to determine the IC_{50}. [b] Percentage of inhibiton (%Inh) employing a 50 μM compound solution. [c] IC_{50} represents the compound concentration necessary to reduce by 50% the ADP formation in the ADP-Glo ™assay. [d] Inhibition constant. [e] ND = not determined. For each compound already published, the corresponding reference is reported. [f] FTrees similarity is computed against the query compound **T187**. Global similarity is derived from local similarities which are listed and color-coded on the right-hand side.

Table S3: Protein-ligand interaction occurrences observed in the three MD replicas for **T125**-AKT1 and **T126**-AKT1 complexes in the time window 100-200 ns. Occurrences higher than 30% are highlighted in green.

		T126			T125		
		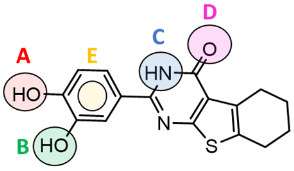			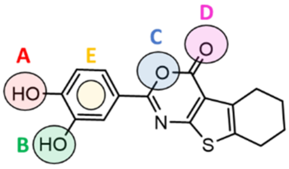		
Protein Residue	***Ligand Group/Atom***	**Replica 1**	**Replica 2**	**Replica 3**	**Replica 1[b]**	**Replica 2**	**Replica 3**
**Ala 230**	A	93%	92%	92%	95%		
**Ala 230**	B				23%	86%[a]	35%[a]
**Glu228**	A	16%	50%	47%			98%
**Glu228**	B				61%[a]	89%	24%
**Thr291**	B	65%	80%	66%			
**Thr291**	C	69%[a]	78%[a]	64%[a]			
**Asp292**	C	73%[a]	80%[a]	72%[a]		10%[a]	
**Asp292**	D	100%[a]	100%[a]	100%[a]		70%[a]	66%[a]
**Leu156**	D				11%[a]		
**Phe438**	E				57%		
**Tyr229**	A				71%[a]		
**Lys179**	D					10%[a]	17%[a]
**Thr211**	B						23%[a]

[a] water-mediated interaction.
[b] only for this replica, the intermolecular interaction occurrences were calculated in the time window 150-200 ns.

Table S4: Composition of Enriched Methocult medium

**Enriched Methocult medium composition**
Methylcellulose in Iscove's MDM
Fetal bovine serum
Bovine serum albumin
2-Mercaptoethanol
Recombinant human interleukin 3 (IL-3)
Recombinant human interleukin 6 (IL-6)
Recombinant human erythropoietin (EPO)
Recombinant human granulocyte colony-stimulating factor (G-CSF)
Recombinant human granulocyte-macrophage colony-stimulating factor (GM-CSF)
Supplements
